# The influence of childhood socioeconomic status on indulgent consumption

**DOI:** 10.3389/fpsyg.2025.1500845

**Published:** 2025-04-16

**Authors:** Chenghu Zhang, Guifeng Meng, Ai Deng

**Affiliations:** School of Economics and Management, Communication University of China, Beijing, China

**Keywords:** childhood socioeconomic status, indulgent consumption, pursuit of pleasure, sense of worthiness, sense of control

## Abstract

**Introduction:**

Consumers routinely confront goal conflicts, navigating the tension between persevering in goal pursuit and succumbing to the temptation of indulgent purchases. While prior literatures have endeavored to explore diverse factors influencing indulgent consumption, they have largely overlooked the longitudinal lens of consumer development to uncover deeper, underlying causes. Childhood socioeconomic background has been established as a critical factor influencing various behaviors both in early life and adulthood. This study aims to explore the impact of childhood socioeconomic status on indulgent consumption in adulthood, and to examine the mediating role of pleasure pursuit in this relationship, as well as the moderating effect of individuals' sense of worthiness.

**Methods:**

Across four behavioral experiments, spanning three domains of indulgence-related decisions and relying on different methods of childhood socioeconomic status measurement, this study sheds light on how consumers of different childhood socioeconomic status to choose when facing a conflict decision (self-control vs. indulgence). A total of 627 participants from China (66.19% female, M_age_ = 30.46) were recruited through the Credamo platform for this study, and SPSS analytical software was utilized to conduct comprehensive analyses on the relevant data, encompassing primary effect analysis, mediation analysis, and moderated mediation analysis.

**Results:**

The study corroborates that individuals with high childhood socioeconomic status are more likely to choose indulgence compared to those with low childhood socioeconomic status (β = 0.24, *p* < 0.05). This effect is found to be independent of people's current level of socioeconomic status (*p* > 0.05), which is mediated by differences in pleasure pursuit [β = 0.1026, 95% CI= (0.0312, 0.1688)]. In other words, individuals who grew up wealthy are generally more likely to pursue pleasure (novel experiences and potential rewards) from decision-making, thus increasing their choice of indulgences. Furthermore, the strength of this effect is moderated by individuals' sense of worthiness (β = 0.15, *p* < 0.05). Lastly, sense of control has been disqualified as a plausible psychological mechanism underlying this phenomenon [95% CI= (−0.035, 0.021)].

**Discussion:**

This study demonstrates that childhood socioeconomic status has a significant positive impact on indulgent consumption in adulthood. The underlying psychological mechanisms and boundary conditions of this influence were also examined. The findings offer a novel theoretical perspective on the antecedents of indulgent consumption and provide valuable insights for businesses in developing targeted marketing strategies and enhancing consumer wellbeing.

## 1 Introduction

In daily life, people often face the dilemma of yielding to or resisting temptation. For example, individuals with weight loss and body-shaping goals must decide whether to adhere to these goals or give in to their desires when faced with a tempting cheesecake (Dholakia et al., 2005; Kokkoris et al., 2019). When people choose to accept the temptation, this behavior, which is pleasure-oriented but often detrimental to their long-term goals, is called indulgent consumption (Salerno et al., 2014). It brings pleasure to consumers while also inducing irrational behaviors, hindering the pursuit of high-value goals, and even leading to harmful behaviors (Kathryn et al., 2008; Yang and Jin, 2018), such as overeating, abusing drugs and alcohol, getting involved in violent conflicts, saying hurtful things to others, overspending, or procrastinating when you have to work (Yang and Jin, 2018).

Current researches on the influencing factors of indulgent consumption can be roughly divided into five categories: personal factors, justifications, product factors, situational factors, and environmental factors (Yang and Jin, 2018; Nenkov and Scott, 2014). Personal factors typically refer to individual traits, such as the level of self-control. Justifications usually refer to the rationalization or legitimization of one's indulgent consumption behavior to relax self-control (Kivetz and Zheng, 2006), such as special anniversaries. Product factors refer to the attributes of the product itself, such as cuteness (Nenkov and Scott, 2014). Situational factors are the contexts in which individuals make decisions, mainly related to decision-making modes, such as single evaluation versus joint evaluation (Fishbach and Zhang, 2008). Environmental factors refer to the environment in which people make decisions, such as ambient lighting (Biswas et al., 2017), and digital modes of ordering in restaurants (Abell et al., 2024).

It is apparent that existing researches mainly remains at the cross-sectional level, lacking attention to longitudinal factors which are more fundamental. This study proposes that childhood socioeconomic status may be an important longitudinal factor influencing indulgent consumption behavior. Childhood socioeconomic status refers to the extent to which individuals grow up in resource-rich or resource-poor environments (Griskevicius et al., 2011). Previous research has indicated that childhood socioeconomic status is an important measure of early childhood experiences, and it can better predict individual behavior patterns and preferences compared to current socioeconomic status (Griskevicius et al., 2011; Thompson et al., 2020). Because consumers tend to influence their future consumption behavior based on their past experience with indulgent opportunities (May and Irmak, 2014). In addition, internal material dissatisfaction and subjective economic insecurity may be the basis for forming the lifelong behavior pattern of consumers (Ahuvia and Wong, 2002; Kasser et al., 1995). However, the extant literature predominantly concentrates on the role of childhood socioeconomic status in the formation of individual traits, such as perceived control (Mittal and Griskevicius, 2014), aggression (Odgers et al., 2008), and altruism (Li et al., 2020). Additionally, discussions pertaining to consumer behavior have been predominantly anchored within the health domain, with an absence of focus on indulgent consumption.

This study seeks to establish a theoretical linkage between childhood socioeconomic status and indulgent consumption. It further aims to investigate how childhood socioeconomic status influences indulgent consumption behaviors in adulthood, while exploring the underlying psychological mechanisms and boundary conditions of this effect. Intuitively, there are inconsistent ideas regarding the relationship between childhood socioeconomic status and indulgent consumption. For example, a deprived living environment in childhood may cause children to develop the instinct to resist temptation in order to maintain survival (Griskevicius et al., 2011), thereby having better resistance to temptation when facing indulgent opportunities. However, childhood deprivation may also lead individuals to perceive indulgent opportunities as rare and precious (Proffitt Leyva et al., 2020), making them feel they should cherish and live in the moment. For individuals with abundant resources in childhood, good education may allow them strong self-control to resist temptation (Mittal and Griskevicius, 2014; Thompson et al., 2020), or they may feel that indulging themselves is justified and not something to endure (Liu et al., 2019). In summary, based on previous research, conflicting inferences have emerged about the impact of childhood socioeconomic status on indulgent consumption. This study attempts to reconcile the above paradox and make an effective supplement to the relevant research. By establishing a theoretical framework linking childhood socioeconomic status to indulgent consumption, this study contributes to a deeper understanding of how childhood experiences influence indulgent consumption choices, helping consumers mitigate unnecessary indulgent behaviors. The findings also offer new insights for businesses involved in the production or sale of indulgent products, providing valuable guidance for more targeted and effective market segmentation and marketing strategies.

### 1.1 Childhood socioeconomic status

Childhood socioeconomic status (CSES) refers to the extent to which people grow up in resource-rich or resource-poor environments (Griskevicius et al., 2011). Childhood is defined as the period of life development from the end of infancy to the onset of adolescence, typically ending at 11 years for girls and 13 years for boys, who have not yet developed secondary sexual characteristics (Lloyd et al., 2014). However, in discussions of CSES, the age boundaries are not strictly adhered to, e.g., Proffitt Leyva et al. (2020), in their study on the effects of CSES on eating behaviors, defined children as being between the ages of 3 and 14 years.

All organisms face a fundamental challenge of successfully allocating time, resources, and energy among the various tasks necessary for survival and reproduction (Griskevicius et al., 2011). The life-history theory emphasizes the fact that people who adopt different life-history strategies to adapt to the conditions of life have specific personality traits that are associated with particular ways of solving trade-offs. Studies have shown that lower CSES is more challenging and unpredictable compared to higher CSES (Griskevicius et al., 2011). Consequently, people growing up with low CSES are likely to adopt fast life-history strategies, whereas those with high CSES tend to adopt slow life-history strategies (Griskevicius et al., 2011). This distinction can affect eating behaviors, as adults who report growing up in relatively safe and resource-rich environment tend to eat based on energy needs, while those from lower socioeconomic backgrounds tend to consume more food regardless of energy requirements (Proffitt Leyva et al., 2020; Miller et al., 2018). It may also influence preferences for immediate versus delayed gratification, e.g., people with higher CSES will attempt to increase future rewards by delaying immediate satisfaction, whereas those from lower CSES will place more value on immediate gains.

People from different CSES backgrounds may develop different control models (Thompson et al., 2020). For example, those from low CSES backgrounds exhibit personal agency by actively adapting to their environment and aligning themselves with it. Interviews with Hurricane Katrina survivors revealed that those who evacuated before the storm were more likely to be middle-class, exercising independence and self-control by choosing to leave, whereas those who stayed were more likely to be working-class, expecting to display resilience and strength in facing challenges rather than give up (Stephens et al., 2009). This also explains why lower CSES consumers are more likely to show patience for their initial choices when consumption options are limited (Thompson et al., 2020). It should be noted that family education models might influence the formation of different agency models (Kusserow, 1999). For instance, an ethnographic study on American family parenting values and strategies showed that middle-class parents emphasize the importance of self-direction and autonomy, whereas working-class and poor parents stress obedience to external authority (Weininger et al., 2009).

The measurement of CSES primarily involves two main subjects: the subjects themselves and their parents. Subjects are often asked to recall their family income during their upbringing (Mittal and Griskevicius, 2014) or to rate three subjective items (Griskevicius et al., 2011), such as “Compared to other children at my school, I felt relatively wealthy.” When using parents' information for measurement, four objective items are generally included: the number of years of education of the father/mother, the father's occupation, and the family income (Zhang et al., 2020). Sometimes, measurements are based solely on the mother's socioeconomic background and educational level (Stamos et al., 2019).

### 1.2 Indulgent consumption

The discussion on the definition of indulgent consumption has matured considerably. Some scholars emphasize that indulgent consumption is a behavior where individuals pursue unnecessary qualities and pleasures (Berry, 1994). Others point out that it is a rapid, unplanned reaction made without considering the negative impact of the stimulus on oneself or others (Moeller et al., 2001). Specifically, Cavanaugh (2014) noted that if consumers regard a purchase as a treat for themselves, any choice can be considered indulgent consumption. Generally, indulgent consumption is widely recognized as a behavior characterized by low self-control and unhealthy consumption (Kahn and Brian, 2004).

Current research on the antecedents of indulgent consumption can be roughly divided into five categories: personal factors, justifications, product factors, situational factors, and environmental factors (Yang and Jin, 2018; Nenkov and Scott, 2014). Personal factors typically refer to individual traits, such as the level of self-control. Justifications usually refer to the rationalization or legitimization of one's indulgent consumption behavior to relax self-control (Kivetz and Zheng, 2006), such as special anniversaries. Product factors refer to the attributes of the product itself, such as cuteness (Nenkov and Scott, 2014). Situational factors are the contexts in which individuals make decisions, mainly related to decision-making modes, such as single evaluation versus joint evaluation (Fishbach and Zhang, 2008). Environmental factors refer to the environment in which people make decisions, such as ambient lighting (Biswas et al., 2017), and digital modes of ordering in restaurants (Abell et al., 2024).

Currently, the measurement of indulgent consumption mainly falls into three categories: entertainment, luxury consumption, and food consumption. In the entertainment category, conflicts between work or study and entertainment are set to highlight the nature of entertainment, such as hanging out with friends or watching TV versus studying (Fishbach and Dhar, 2005; Kivetz and Zheng, 2006), playing games versus completing tasks (O'Brien and Roney, 2017). In the luxury consumption category, the emphasis is on people with limited funds and long-term savings goals consuming expensive goods beyond their purchasing power, such as buying a coveted but expensive sweater with limited funds (Dholakia et al., 2005; Kokkoris et al., 2019). Food consumption is the most common measurement of indulgent consumption. Typically, people with long-term goals of maintaining a figure and health or with short-term weight loss and body-shaping aspirations face the temptation of high-calorie foods (Dholakia et al., 2005; Kokkoris et al., 2019). For example, those in need of weight loss have to choose between high-calorie but tempting chips and a bland vegetable salad (Dholakia et al., 2005), or allowing subjects to eat chips while completing experimental tasks and then calculating the chip consumption.

In summary, most scholars view indulgent consumption behavior from a cross-sectional perspective, lacking attention to longitudinal factors. Vanbergen and Laran (2016) pointed out that childhood experiences might impact self-regulation abilities which are closely related to indulgent consumption. Therefore, longitudinal factors may indeed influence indulgent consumption behavior, but research in this area has yet to be fully explored.

### 1.3 The main effect of childhood socioeconomic status on indulgent consumption

Material conditions shape different agency models, providing a guide for how to think, feel, and act (Carey and Markus, 2016; Markus and Conner, 2013). Early resource scarcity can limit an individual's ability to change their environment (Infurna et al., 2011). Research confirms that people from poor families may be unable to fundamentally control stressful events and may therefore give up efforts to solve such events (Park et al., 2022). In such environments, individuals with low CSES may learn an agency model that emphasizes self-control rather than environmental control (Thompson et al., 2020). Conversely, individuals with high CSES, who have more resources to manage stressful events, are more likely to view personal agency in terms of autonomy and making proactive choices (Markus and Conner, 2013; Stephens et al., 2009), i.e., they tend to demonstrate internal demand and exert control by aligning the environment with their desires (Stephens et al., 2009).

Additionally, Kusserow's (1999) study shows that high SES (upper-middle-class) parents respect their children's emotions and desires, enabling them to find the right social path for themselves (i.e., developing primary control). In contrast, low SES (working-class) parents believe that children need to learn that they cannot always get what they want and must toughen up (i.e., developing secondary control). Relatedly, studies indicate that disadvantaged children score higher on inhibition and problem-solving than their non-disadvantaged peers (Ibane-Aonso et al., 2021). Moreover, individuals with low socioeconomic status are not more impulsive or shortsighted. When choices are unavailable, they exhibit more patience (Thompson et al., 2020). It is also noteworthy that Luthar and D'Avanzo (1999) found that students from affluent families in large American cities were more likely to engage in negative behaviors such as smoking, drinking, and using marijuana compared to their peers from impoverished urban areas.

Therefore, when faced with self-conflict due to indulgent opportunities, individuals with low CSES are more likely to adopt secondary control, while those with high CSES tend to take control into their own hands. Thus, we hypothesize that:

*H1*: Individuals with relatively high (vs. low) childhood socioeconomic status show stronger preference for indulgent consumption.

### 1.4 The mediating effect of the pursuit of pleasure

Previous research shows that individuals with lower SES (compared to those with higher SES) have more perceived constraints, greater feelings of helplessness, and less self-direction. Additionally, individuals who grew up in stressful, resource-scarce environments may perceive the future as uncertain and people around them as untrustworthy (Li et al., 2020). Consequently, they are more cautious, skeptical, and vigilant toward external stimuli (Stamos et al., 2019). They are less likely to be novel to the outside world and will feel relatively more constrained in their actions than high CSES individuals.

Unlike low-CSES individuals, high-CSES individuals are taught from a young age that they are independent, unique, and they can control their destiny, even shaping the world around them (Stephens et al., 2009). They are also more inclined to please themselves and seek variety (Zhao et al., 2022). Liu et al. (2019) confirmed that the richer a person's upbringing, the more likely they are to develop dispositional greed. When having grown accustomed to the relatively abundant resources of their childhood, people may feel entitled to desire more. Thus, compared to low CSES individuals, high CSES individuals are more likely to seek out and approach rewards or novel experiences.

The pursuit of pleasure reflects motivation to seek novel rewards (Chen et al., 2015), emphasizing the desire for new rewards and reflecting a willingness to approach potential reward events impulsively (Dambrun and Ricard, 2011). Therefore, compared to low CSES individuals, high CSES individuals are more likely to desire rewards and have a higher willingness to approach potential reward events. Thus, this paper proposes the following hypothesis:

*H2*: Individuals with relatively higher (vs. low) childhood socioeconomic status demonstrate stronger pursue of pleasure.

In daily life, choices between hedonic and utilitarian products constitute a frequent trade-off decision closely (Yang and Jin, 2018). Virtue is closely associated with goals and self-control, while motivation helps individuals maintain self-control to some extent (Yang and Jin, 2018). Thus, compared to highly motivated individuals, those with lower motivation to maintain self-control are less likely to sustain self-control. The pursuit of pleasure primarily relies on external stimuli (Dambrun and Ricard, 2011), reflecting the willingness to approach potential reward events impulsively (Dambrun and Ricard, 2011). Therefore, when indulgent events become external stimuli and choosing indulgence can yield rewards, individuals with a high pursuit of pleasure are relatively more likely to approach indulgent events impulsively compared to those with a low pursuit of pleasure. In other words, in indulgent situations, individuals with a higher willingness to pursue pleasure are more likely to choose indulgence. Therefore, this paper proposes the following hypotheses:

*H3*: The pursuit of pleasure positively influences individual's preference for indulgent consumption.

*H4*: The pursuit of pleasure mediates the effect of childhood socioeconomic status on indulgent consumption intention.

### 1.5 The moderating effect of a sense of worthiness

Prior research has shown that consumers will experience feelings of guilt when they choose an indulgence or hedonic goods due to their inability to resist temptation (Yang and Jin, 2018) and that justifying reasons can defuse such feelings (Yang and Jin, 2018). The justification here refers to consumers making excuses for their inconsistent behavior in order to accept the expected failure before the indulgence is actually performed (Huberts et al., 2014). But wanting to do something is only a prerequisite for action (Huberts et al., 2014), consumers must also feel worthy of doing it (Miller and Effron, 2010). Thus, “a sense of worthiness,” such as “I've worked hard all day, I deserve a reward” (Taylor et al., 2014), can be used as a form of justification (Heiland and Veilleux, 2022).

Justification focuses the consumers' attention on the temptation and increases the value of the temptation by giving them a rationale about why it is reasonable to give in to the temptation (Heiland and Veilleux, 2022). Thus, when an individual possesses a higher sense of worthiness, the more valuable the temptation is in their eyes. In other words, individuals with a high sense of worthiness should have a greater willingness to indulge relative to individuals who possess a low sense of worthiness. Thus, when the sense of worthiness is high, individuals with high childhood socioeconomic status should be more likely to enhance their indulgent consumption intention. Whereas, when the sense of worthiness is low, indulgences are less valuable in the eyes of the consumers, and their intention to consume indulgences should not be significantly greater regardless of socioeconomic status.

Meanwhile, when the positive effect of pleasure pursuit on indulgent consumption intention holds true, the influence of pleasure pursuit on indulgent consumption intention should be stronger as the sense of worthiness rises. In summary, the following hypothesis is proposed:

*H5*: Sense of worthiness positively moderates the direct effect of childhood socioeconomic status on indulgent consumption. The positive effect of childhood socioeconomic status on the indulgent consumption is significantly stronger (weaker) when the sense of worthiness is higher (lower).

*H6*: Sense of worthiness positively moderates the mediating effect of pleasure pursuit between childhood socioeconomic status and indulgent consumption intention. The mediating effect of pleasure pursuit between childhood socioeconomic status and indulgent consumption is stronger (weaker) when a sense of worthiness is higher (lower).

**Figure 1:**
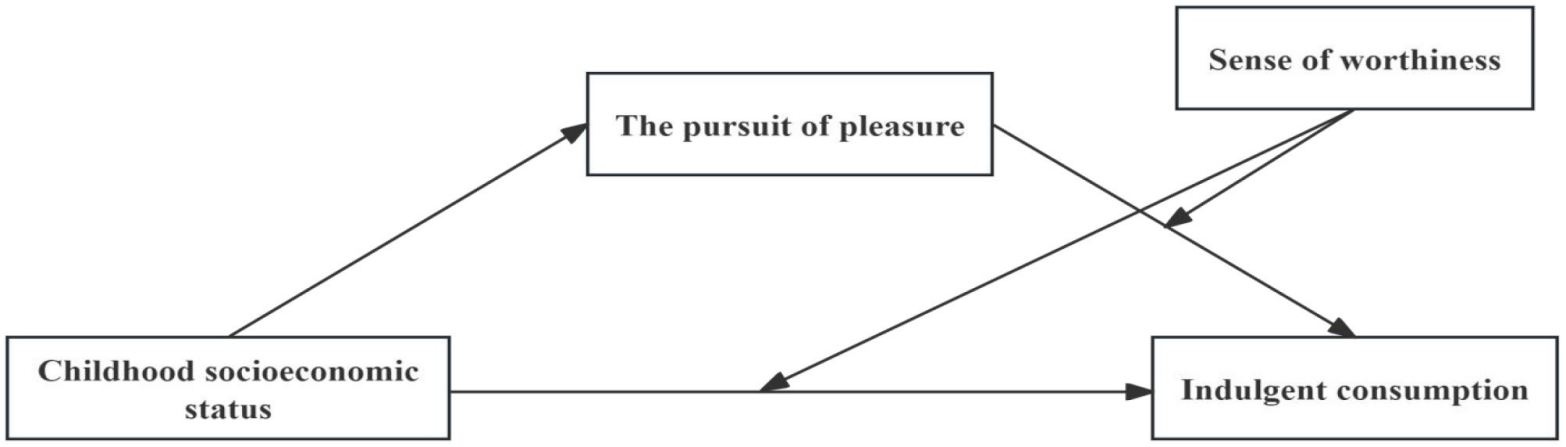
Hypothesized model.

Overall, drawing from both theoretical and empirical perspectives, this study constructed a moderated mediation model (see Figure 1).

## 2 Materials and methods

### 2.1 Study 1: showing the main effect of childhood socioeconomic status

The aim of this study was primarily to test Hypothesis 1. That is, individuals with higher childhood socioeconomic status are more inclined to indulge in situations of indulgence compared to those with lower childhood socioeconomic status.

#### 2.1.1 Participants

This study was conducted in China and received approval from the Ethics Committee of the School of Economics and Management at Communication University of China. In Study 1, a total of 130 participants were recruited through the Credamo platform, with one participant failing the attention check, resulting in 129 valid samples (69.0% female, Mage = 31). Prior to the experiment, all participants read an informed consent form, which assured them that their responses would be kept confidential and anonymous. The data collected were solely for academic research purposes and held no commercial intent. Participants were free to join or withdraw from the study at any time. All participants who completed the experimental tasks received a cash reward.

#### 2.1.2 Measures

(1) Indulgent consumption. Food choice decisions are among the most common contexts used to measure indulgent consumption. Drawing on the studies by Dholakia et al. (2005) and Kokkoris et al. (2019), Study 1 assessed participants' willingness to indulge in high-calorie foods while pursuing weight loss goals. Participants were asked to imagine and decide to what extent they would be willing to consume indulgent foods under the following scenario: “After finishing work on a weekday, you plan to visit a shopping mall to relax. Currently, you are aiming to lose the weight you have recently gained and want to avoid foods that are high in fat and sweets. Therefore, you strive to eat healthily and steer clear of high-calorie foods in order to achieve your weight loss and body shaping goals. However, as you pass through the shopping center, you walk by your favorite dessert shop in the food court. As you glance through the food display cabinet, you are immediately drawn to a plate of mouth-watering strawberry cheesecake, your favorite dessert. Its sweet taste and beautiful appearance tempt you, and you feel a strong desire to eat it right away. Since you do not visit this mall often due to its distance from your location, you are unlikely to return to this dessert shop in the near future.” Indulgent consumption was measured with the following question: “To what extent would you be willing to purchase this strawberry cheesecake?” Responses were recorded on a 7-point Likert scale (1 = Not at all, 7 = Extremely).

(2) Childhood socioeconomic status. The measurement of childhood socioeconomic status was adapted from the scale used by Griskevicius et al. (2011), which consists of three items (e.g., “During my childhood, my family usually had enough money to buy a variety of things,” “I grew up in a relatively affluent environment,” “Compared to other children at school, I felt relatively wealthy”). Participants were asked to indicate their level of agreement with these statements on a 7-point Likert scale (1 = Strongly disagree, 7 = Strongly agree). The scale demonstrated good reliability (Cronbach's α = 0.957), indicating high internal consistency and suitability for the sample in Study 1.

(3) Current socioeconomic status. The measurement of current socioeconomic status was adapted from the scale used by Griskevicius et al. (2011), which includes three items (e.g., “I have enough money to buy the things I want,” “I don't need to worry too much about paying my bills,” “I feel that I won't have to worry about money in the future”). Participants were asked to indicate their level of agreement with these statements on a 7-point Likert scale (1 = Strongly disagree, 7 = Strongly agree). The scale demonstrated good reliability (Cronbach's α = 0.914), indicating high internal consistency and suitability for the sample in Study 1.

#### 2.1.3 Procedure

Study 1 employed a within-subjects design with a single factor. Initially, all recruited participants were informed: “You are about to participate in a “consumer choice” survey. There are no right or wrong answers to the questions in the questionnaire; please respond according to the given scenarios.” Participants were then asked to read a passage and imagine themselves in the situation to make a consumption decision (“To what extent would you be willing to consume high-calorie foods while pursuing weight loss goals?”; Dholakia et al., 2005; Kokkoris et al., 2019). Subsequently, participants were asked to rate the extent to which they agreed with the statement, “Purchasing the strawberry cheesecake at this moment is an indulgent behavior” (1 = Strongly disagree, 7 = Strongly agree), to assess the validity of the indulgent consumption scenario. Finally, participants completed the childhood socioeconomic status scale, the current socioeconomic status scale, and provided demographic information such as gender and age. The order of items in the scales was presented randomly.

#### 2.1.4 Data analysis

Study 1 used SPSS 24.0 for data analysis. The validity of the indulgent consumption scenario was assessed using a one-sample *t*-test, testing whether the sample mean was significantly >4. The effect of childhood socioeconomic status on indulgent consumption (main effect analysis) was analyzed using a general linear regression.

#### 2.1.5 Results

(1) Manipulation check. The results of the one-sample *t*-test indicated that participants' perception of indulgence associated with purchasing a strawberry cheesecake was significantly greater than the median value (M = 5.31, SD = 1.12, *p* < 0.001), suggesting successful manipulation.

(2) Main effect analysis. Using childhood socioeconomic status as the independent variable and indulgent consumption intention as the dependent variable, we conducted linear regression analyses. The results showed that CSES had a significant main effect (β = 0.15, *p* < 0.05). Then, incorporating current socioeconomic status into the linear model revealed that the main effect of childhood socioeconomic status remained significant (β = 0.24, *p* < 0.05), while current socioeconomic status had no significant effect on indulgent consumption intention (β = −0.15, *p* > 0.05). Finally, incorporating gender and age into the linear model, the analysis revealed that none of these variables significantly affected individual indulgent consumption intention (*p*_gender_ = 0.283, *p*_age_ = 0.319). Therefore, childhood socioeconomic status is a better predictor of indulgent consumption intention than current socioeconomic status, and childhood socioeconomic status had a significantly positive effect on indulgent consumption intention, thus supporting H1.

#### 2.1.6 Discussion

Study 1 confirms that childhood socioeconomic status has a positive effect on indulgent consumption intention and that this effect cannot be replaced by current socioeconomic status. This is in line with the findings of Griskevicius et al. (2011). Individuals' willingness to seek pleasurable experiences and approach potentially rewarding events is correlated with the dimension of pleasure pursuit in the behavior-activating system. Therefore, in the next experiment, further research will be conducted to investigate how childhood socioeconomic status influences indulgent consumption behavior, examining whether differences in resource abundance in childhood affect pleasure pursuit and thus their indulgent consumption behavior.

### 2.2 Study 2: showing the mediating effect of the pursuit of pleasure

The aim of this study was to validate the robustness of the main effect identified in Study 1 and to corroborate Hypotheses 2–4, specifically, examining the mediating role of pleasure in the influence of childhood socioeconomic status on indulgent consumption.

#### 2.2.1 Participants

This study was conducted in China and received approval from the Ethics Committee of the School of Economics and Management at Communication University of China. In Study 2, a total of 145 participants were recruited through the Credamo platform, with one participant failing the attention check, resulting in 129 valid samples (69.0% female, Mage = 31). Prior to the experiment, all participants read an informed consent form, which assured them that their responses would be kept confidential and anonymous. The data collected were solely for academic research purposes and held no commercial intent. Participants were free to join or withdraw from the study at any time. All participants who completed the experimental tasks received a cash reward.

#### 2.2.2 Measures

(1) Indulgent consumption. Building on the studies by Dholakia et al. (2005) and Kokkoris et al. (2019), Study 2 asked participants to imagine and decide their willingness to purchase shoes under a limited budget. The decision-making scenario was as follows: “You plan to visit the mall to buy a few pairs of socks. Due to a tight budget, you do not wish to spend money on any other items, and you currently do not need anything else. However, as you walk through the mall, your attention is immediately drawn to a stylish and attractive pair of shoes. These shoes happen to be in your size, and their color, design, and style are to your liking, making them look great on you. The salesperson informs you that this is the last pair available, and they are unlikely to continue selling this style in the future. Despite this, the shoes are expensive and unnecessary at the moment.” After reading this scenario, participants were asked to rate their willingness to engage in indulgent consumption (“To what extent are you willing to purchase these shoes at this moment?”), using a 7-point Likert scale (1 = Not at all, 7 = Extremely).

(2) Childhood socioeconomic status and current socioeconomic status. In Study 2, the measurements of childhood socioeconomic status and current socioeconomic status followed the same approach as in Study 1, utilizing the scales developed by Griskevicius et al. (2011). Each scale consisted of three items, and both scales were subjected to reliability testing (Cronbach's α_CSES_ = 0.947, Cronbach's α_SES_ = 0.937).

(3) Pursuit of pleasure. The pursuit of pleasure reflects an individual's willingness to approach potential rewarding events impulsively (Dambrun and Ricard, 2011). Drawing on the description of the “Pursuit of Pleasure” dimension in the behavioral activation scale by Li et al. (2008), Study 2 measured the pursuit of pleasure using five items (e.g., “I often do things just because I find them fun or interesting,” “I enjoy excitement and novelty,” etc.). Participants were asked to indicate their level of agreement with these statements (1 = Strongly disagree, 7 = Strongly agree). The scale demonstrated satisfactory reliability, with a Cronbach's α of 0.862.

#### 2.2.3 Procedure

Study 2 employed a within-subjects design. Participants were first asked to read a scenario and imagine themselves in the situation, making a consumption decision (“willingness to purchase shoes under a limited budget”). They were then asked to what extent they agreed with the statement, “Purchasing the shoes at this moment is an indulgent behavior” (1 = Strongly disagree, 7 = Strongly agree), in order to assess the validity of the indulgent consumption scenario. Next, participants completed the pursuit of pleasure scale. Finally, they filled out the childhood socioeconomic status and current socioeconomic status scales, followed by demographic information such as gender and age.

#### 2.2.4 Data analysis

Study 2 also utilized SPSS 24.0 for data analysis. Similar with Study 1, the validity of the indulgent consumption scenario was assessed using a one-sample *t*-test, while the effect of childhood socioeconomic status on indulgent consumption was analyzed using a general linear model (GLM). The mediating effect of the pursuit of pleasure was examined using the bootstrap method (Model 4, with a sample size of 5,000 and a 95% bias-corrected confidence intervals).

#### 2.2.5 Results

(1) Manipulation check. The results of the one-sample *t*-test indicated that participants' perception of indulgence associated with buying these shoes was significantly greater than the median value (M = 5.78, SD = 1.205, *p* < 0.001), suggesting successful manipulation.

(2) Main effect analysis. Using childhood socioeconomic status as the independent variable and indulgent consumption as the dependent variable, regression analysis showed that childhood socioeconomic status had a significant positive effect on indulgent consumption (β = 0.58, *p* < 0.001). Incorporating current socioeconomic status into the model revealed that the main effect of childhood socioeconomic status remained significant (β = 0.61, *p* < 0.001), while current socioeconomic status had no significant effect on indulgent consumption intention (β = 0.62, *p* > 0.05). Adding variables such as gender, and age into the model, the main effect of childhood socioeconomic status remained significant *(*β = 0.62, *p* < 0.001). Gender had a significant negative effect on indulgent consumption intention (β = 0.60, *p* < 0.001), meaning that compared to males, females had a stronger intention toward indulgent consumption. Age had a significant positive effect on indulgent consumption intention (β = 0.05, *p* < 0.001), indicating that the older the individual, the stronger the intention toward indulgence. Current socioeconomic status had no significant effect on indulgent consumption intention (β_SES_ = −0.129, *p* > 0.05).

(4) Mediation analysis. For the independent variable (childhood socioeconomic status) and the dependent variable (indulgent consumption intention), Model 4 was selected with a sample size of 5,000 and a 95% confidence interval. Bootstrap-mediated tests showed that childhood socioeconomic status had a significant positive effect on pleasure pursuit (β = 0.19, *p* < 0.001). Pleasure pursuit had a significant positive effect on indulgent consumption intention (β = 0.55, *p* < 0.001), supporting hypotheses 2 and 3. As shown in Table 1, the mediation effect of pleasure pursuit was significant (BootLLCI = 0.0312, BootULCI = 0.1688, 95% CI), partially mediating (BootLLCI = 0.3401, BootULCI = 0.6171, 95% CI), supporting hypothesis 4.

**Table 1:** Mediating effects of pleasure pursuit.

**Path**	**β**	**LLCI (BootLLCI)**	**ULCI (BootULCI)**
Direct effect of X on Y	0.4786	0.3401	0.6171
indirect effect of X on Y	0.1026	0.0312	0.1688

#### 2.2.6 Discussion

Study 2 confirmed that pleasure pursuit mediated the positive effect of childhood socioeconomic status on indulgent consumption. It is important to note that in both Study 1 and Study 2, the measurement of individuals' willingness to engage in indulgent behavior was contextualized within a purchasing decision scenario. Whether the hypotheses remain valid when applied to indulgent behaviors in non-consumption contexts still requires further investigation. Study 3 will address this by varying the scenarios of indulgent decision-making, aiming to enhance the generalizability of the conclusions drawn from Hypotheses 1 and 2 and explore their potential boundary conditions.

### 2.3 Study 3: showing the moderating effect of a sense of worthiness

The aim of this study was to validate the moderating role of a sense of worthiness in the influence of pleasure pursuit on indulgent consumption behavior (Hypotheses 5–6).

#### 2.3.1 Participants

This study was conducted in China and received approval from the Ethics Committee of the School of Economics and Management at Communication University of China. In Study 3, a total of 189 participants were recruited through the Credamo platform, with 179 valid responses collected (68.2% female, Mage = 30.35). Prior to the start of the experiment, all participants read the informed consent form and were informed of their right to either participate or withdraw from the study at any time. Participants who completed the experimental tasks received a monetary reward.

#### 2.3.2 Measures

(1) Indulgent consumption. Building on the work of Fishbach and Dhar (2005), Study 3 tests indulgent behavior by having participants make a decision between studying at home and going out with friends to watch a movie. The decision scenario is presented as follows: “It is 3 PM, and you are diligently studying in your study room in preparation for an important exam tomorrow afternoon. Coincidentally, today is the premiere of a movie you have been eagerly anticipating, and a friend has invited you to join them for the screening at 7 PM. Prior to the release, the movie's promotional posters have been everywhere, featuring your favorite film stars. According to media reports, the movie is well-produced, visually stunning, and has a gripping, fast-paced storyline. You have long been intrigued by this film, but you are also aware that you still have a lot of studying to do. Watching the movie now would take up 3–4 h of your study time, potentially affecting your exam performance.” After reading this, participants are asked to indicate their preference for the decision scenario (1 = strongly prefer to continue studying, 7 = strongly prefer to buy tickets for the movie, 4 = uncertain).

(2) Childhood socioeconomic status and current socioeconomic status. In Study 3, the measurements of childhood and current socioeconomic status followed the same approach as in Studies 1 and 2. The scales were again subjected to reliability testing (Cronbach's α_CSES_ = 0.911, Cronbach's α_SES_ = 0.876).

(3) Pursuit of pleasure. The measurement of the pursuit of pleasure in Study 3 followed the same scale used in Study 2, consisting of five items. The scale was again subjected to reliability testing, yielding a Cronbach's α of 0.883.

(4) Sense of worthiness. Drawing on the work of Taylor et al. (2014) and Cavanaugh (2014), the measure of self-worth consisted of five items (e.g., “I believe I deserve to be treated well,” “I believe I deserve to be rewarded,” “I believe I deserve to be pleased,” “I believe I should be treated well,” “I believe I need to be treated well”). Participants were asked to indicate their level of agreement with each statement (1 = Strongly disagree, 7 = Strongly agree). The scale demonstrated good reliability, with a Cronbach's α of 0.962.

#### 2.3.3 Procedure

Study 3 employed a within-subjects design (childhood socioeconomic status × sense of worthiness). Participants were first asked to read a scenario and imagine themselves making a decision between studying at home and going out with friends to watch a movie. They were then asked to indicate the extent to which they agreed that “spending 3–4 h of study time watching a movie” constitutes indulgent behavior (1 = Strongly disagree, 7 = Strongly agree), in order to assess the effectiveness of the experimental manipulation. Next, participants completed the pursuit of pleasure scale and the sense of worthiness scale. Finally, they filled out the childhood socioeconomic status scale, the current socioeconomic status scale, and demographic information such as gender and age.

#### 2.3.4 Data analysis

Data analysis for Study 3 was conducted using SPSS 24.0. The effectiveness of indulgent material selection was assessed using a one-sample *t*-test. The analysis of moderated mediation effects was conducted through a three-step regression approach and the bootstrap method (Model 14), with a sample size of 5,000 and a 95% bias-corrected confidence interval.

#### 2.3.5 Results

(1) Manipulation check. The results of the one-sample *t*-test indicated that participants' perception of indulgence associated with watching the movie was significantly greater than the median value (M = 5.75, SD = 1.102, *p* < 0.001), suggesting successful manipulation.

(2) Moderated mediation analysis. Following the steps outlined by Wen and Ye (2014) for testing a moderated mediation model: in the first step, the interaction between childhood socioeconomic status and the sense of worthiness was significantly correlated with indulgent consumption intention (β = 0.15, *p* < 0.05), as shown in Table 2. It indicated that the sense of worthiness significantly moderated the direct effect of childhood socioeconomic status on indulgent consumption intention, supporting hypothesis 5. In second step, childhood socioeconomic status has a significant positive effect on pleasure pursuit (β = 0.22, *p* < 0.05). The mediating effect of pleasure pursuit is significant (β = 0.75, *p* < 0.001), but the interaction term between pleasure pursuit and the sense of worthiness is not significant (β = 0.16, *p* = 0.075). In the third step, following the suggestion of Wen and Ye (2014), when none of the pairs of coefficients in the sequential testing are significant (i.e., no significant interaction), the non-parametric percentile Bootstrap method can be used for interval testing.

**Table 2:** Testing the moderated mediation effect.

**Predictors**	**Model 1 (ICI)**		**Model 2 (PP)**		**Model 3 (ICI)**	
	**β**	**t**	**β**	**t**	**β**	**t**
CSES	0.26^*^	2.23	0.22^**^	3.10	0.09	0.84
PP					0.7^***^	6.75
SW	0.34^**^	2.82	0.24^**^	3.16	0.23^*^	1.98
CSES × SW	0.15^**^	2.35			0.09	1.44
SW × PP					0.16	1.80
Age	0.01	0.39	−0.01	−1.00	0.02	1.11
Gender	−0.49	−1.62	−0.11	−0.61	−0.37	−0.16
SES	0.00	0.00	0.59	−0.85	0.02	0.19
*R* ^2^	0.15		0.13		0.35	
F	4.17^**^		4.17^***^		10.13^***^	

^*^p < 0.05, ^**^p < 0.01, ^***^p < 0.001. CSES, childhood socioeconomic status; PP, pursuit of pleasure; SW, sense of worthiness; ICI, indulgent consumption intention; SES, current socioeconomic status.

Using childhood socioeconomic status as the independent variable, pleasure pursuit as the mediating variable, the sense of worthiness as the moderating variable, and indulgent consumption intention as the dependent variable, controlling for gender, age, and current socioeconomic status, SPSS PROCESS macro plugin is applied for Bootstrap method testing, selecting Model 14, with a sample size of 5,000 and a 95% confidence interval. The results show that childhood socioeconomic status significantly positively predicts pleasure pursuit (β = 0.19, *p* < 0.001); Pleasure pursuit significantly positively predicts their consumption intention (β = −0.17, *p* < 0.001); The effect of childhood socioeconomic status on indulgent consumption is not significant (β = 0.10, *p* = 0.23); The interaction between pleasure pursuit and sense of worthiness significantly positively predicts indulgent consumption intention (β = 0.19, *p* < 0.001), confirming hypothesis 6.

#### 2.3.6 Discussion

Study 3 developed a moderated mediation model, confirming that a sense of worth positively moderates the mediating effect of the pursuit of pleasure between childhood socioeconomic status and indulgent consumption intention. Specifically, the higher an individual's sense of worth, the stronger the mediating effect of the pursuit of pleasure between childhood socioeconomic status and indulgent consumption, and conversely, the weaker it becomes. However, similar with the first two studies, Study 3 relied on self-reports to assess childhood socioeconomic status, which is highly subjective. Study 4 aims to enhance the robustness of the findings by employing a more objective measurement approach. Additionally, previous research has shown that individuals with high (vs. low) socioeconomic status possess higher sense of control (Mittal and Griskevicius, 2014), and indulgent consumption is highly correlated with self-control. In Study 4, we will also consider the potential for control as an alternative explanatory mechanism.

### 2.4 Study 4: ruling out alternative explanations for sense of control

The primary objective of this study was to reinforce the experiment's robustness by supplementing it with an objective measurement of childhood socioeconomic status. Furthermore, we aimed to eliminate the potential confounding effect of a sense of control as an alternative explanatory mechanism for the influence of childhood socioeconomic status on indulgent consumption behaviors.

#### 2.4.1 Participants

This study was conducted in China and received approval from the Ethics Committee of the School of Economics and Management at Communication University of China. A total of 200 participants were recruited through the Credamo platform for Study 4, with 182 valid samples obtained (63.5% female, Mage = 30.01). Prior to the commencement of the experiment, all participants read and signed an informed consent form, granting them the freedom to participate or withdraw from the study at any time. All participants who completed the experimental tasks received a monetary compensation.

#### 2.4.2 Measures

(1) Indulgent consumption. In Study 4, the measurement of indulgent consumption followed the same approach as in Study 2: participants were asked to read a shopping scenario description and then report their willingness to purchase shoes under a limited budget using a 7-point Likert scale.

(2) Childhood socioeconomic status and current socioeconomic status. In Study 4, the measurements of childhood and current socioeconomic status followed the same approach as in Studies 1 and 2. The scales were again subjected to reliability testing (Cronbach's α_CSES_ = 0.911, Cronbach's α_SES_ = 0.876).

(3) Pursuit of pleasure. The measurement of the pursuit of pleasure in Study 4 followed the same scale used in Study 2, consisting of five items. The scale was again subjected to reliability testing, yielding a Cronbach's α of 0.883.

(4) Sense of control. Sense of control is the belief that an individual has the capability to shape his or her life. The five-item scale used to measure the sense of control was adapted from Mittal and Griskevicius (2014). Participants indicated their agreement with the following statements (Cronbach's α = 0.718): (a) The events in my life are largely determined by my own efforts. (b) Whatever happens in my life is often beyond my control. (c) Whether I can achieve what I desire depends entirely on my own actions. (d) What happens to me in the future is primarily up to me. (e) I have little control over most of the things that occur in my life. Responses for each item were provided on a 7-point scale (1 = strongly disagree, 7 = strongly agree).

#### 2.4.3 Procedure

Study 4 employed a within-subjects design with a single factor. Initially, participants were required to read a passage (identical to the one in Study 2) and imagine themselves in a scenario where they had to make a decision about purchasing shoes within a limited budget. To test the effectiveness of the indulgence manipulation, participants were asked to what extent they agreed with the statement, “Purchasing shoes in this situation is an act of indulgence” (1 = Strongly disagree, 7 = Strongly agree). Subsequently, participants completed both the Pursuit of Pleasure scale and the Sense of Control scale, with the order of items within each scale randomized. Finally, participants provided responses on their subjective childhood and current socio-economic status scales, followed by demographic information such as gender, age, and their parents' occupational and educational backgrounds.

#### 2.4.4 Data analysis

Data analysis for Study 4 was conducted using SPSS 24.0. Consistent with Studies 1–3, the validity of the indulgence manipulation was tested using a one-sample *t*-test. The effect of childhood socioeconomic status on indulgent consumption was analyzed using a general linear model (GLM). The mediating effects of the pursuit of pleasure and sense of control were examined through the bootstrap method (Model 4), with a sample size set to 5,000 and a 95% bias-corrected confidence interval.

#### 2.4.5 Results

##### 2.4.5.1 Manipulation check

The results of the one-sample *t*-test indicated that participants' perception of indulgence associated with buying the shoes was significantly greater than the median value (M = 5.26, SD = 1.507, *p* < 0.001), suggesting successful manipulation.

##### 2.4.5.2 Main effects analysis

Based on the results in Table 3, after controlling for gender, age, and SES, it is evident that objective childhood socioeconomic status significantly positively influences indulgent consumption intention (β = 0.20, *p* < 0.05), thus supporting H1. It is noteworthy that in Model 1, current socioeconomic status significantly positively influences indulgent consumption intention (β = 0.10, *p* < 0.05). However, when objective childhood socioeconomic status enters Model 2, this effect becomes non-significant (β = 0.06, *p*>0.05), thus validating that childhood socioeconomic status is more predictive of indulgent consumption intention compared to current socioeconomic status.

**Table 3:** Direct effect regression analysis of study 4.

**Predictors**	**Model 1 (ICI)**		**Model 2 (ICI)**	
	β	**t**	β	**t**
OCSES			0.20^*^	2.05
Age	−0.05	−0.68	−0.06	−0.82
Gender	−0.10	−1.39	−0.10	−1.27
SES	0.10^*^	2.18	0.06	1.13
*R* ^2^	0.04		0.06	
F	1.76		2.74^*^	

^*^p < 0.05. OCSES, objective childhood socioeconomic status; ICI, indulgent consumption intention; SES, current socioeconomic status.

##### 2.4.5.3 Mediation analysis

Mediation effects of pleasure pursuit in the process of subjective childhood socioeconomic status influencing indulgent consumption intention were examined using the Bootstrap method, with Model 4 selected, and a confidence interval set at 95%. As shown in Table 4, the results indicate that even after controlling for participants' family education background (i.e., father's and mother's education levels), the mediation effect of pleasure pursuit remains significant (BootLLCI = 0.015, BootULCI = 0.240, 95%CI), acting as a complete mediator (LLCI = −0.003, ULCI = 0.320, 95%CI), thus supporting H4.It is worth noting that when examining the mediation effect of sense of control in the process of subjective childhood socioeconomic status influencing indulgent consumption intention using the Bootstrap method, the mediation effect of sense of control is not significant (BootLLCI = −0.035, BootULCI = 0.021, 95%CI). Furthermore, when the sense of control is included as a control variable, the mediation effect of pleasure pursuit remains significant (BootLLCI = 0.035, BootULCI = 0.258, 95%CI), acting as a complete mediator (LLCI = −0.017, ULCI = 0.307, 95%CI).

**Table 4:** Mediating analysis of pleasure pursuit in study 4.

**Predictors**	**Model 1 (PP)**			**Model 2 (ICI)**		
	β	**t**	**95% CI**	β	**t**	**95% CI**
Gender	−0.25	−1.24	[−0.64, 0.14]	−0.13	−0.59	[−0.56, 0.30]
Age	−0.02	−1.41	[−0.65, 0.15]	0.003	0.24	[−0.02, 0.03]
Paternal education	−0.27	−1.72	[−0.58, 0.04]	0.13	0.80	[−0.20, 0.46]
Maternal education	0.22	1.62	[−0.05, 0.50]	0.002	0.01	[−0.29, 0.29]
SCSES	0.20^**^	4.71	[0.34, 0.30]	0.16	1.93	[−0.003, 0.32]
PP				0.63^***^	7.81	[0.47, 0.79]
*R* ^2^	0.11			0.33		
F	3.55^**^			12.49^***^		

^**^p < 0.01, ^***^p < 0.001. SCSES, subjective childhood socioeconomic status; PP, pursuit of pleasure; ICI, indulgent consumption intention. Each variable in the model was standardized.

#### 2.4.6 Discussion

Study 4 used parents' occupation and education level as objective measures of participants' childhood SES and confirmed the positive impact of childhood SES on indulgent consumption behaviors in adulthood, providing further support for the primary hypothesis of this study. Additionally, the potential psychological mechanism of perceived control was excluded as an explanation for this effect. It is important to note that the pursuit of pleasure only mediated the effect of subjective childhood SES on indulgent consumption, while no mediating effect was found when considering the influence of objective childhood SES on indulgent consumption. We speculate that individuals are more likely to form attitudes and behaviors based on their subjective perception of environmental resources, suggesting that, compared to the objective availability of resources, individuals tend to increase their pursuit of pleasure when they perceive themselves as having abundant resources. However, this speculation requires further validation.

## 3 General discussion

Existing research on the factors influencing indulgent consumption typically categorizes them into five types: personal factors, justifications, product factors, situational factors, and environmental factors (Yang and Jin, 2018; Nenkov and Scott, 2014). However, few studies have explored the impact of individuals' developmental environments on indulgent consumption from a longitudinal perspective. This study constructs a framework model to examine the effect of childhood socioeconomic status on indulgent consumption, based on theoretical reasoning, and proposes its underlying psychological mechanisms and boundary conditions. Through four experimental scenarios, six hypotheses were tested, and the findings offer valuable insights for both academic research and business practice in related fields.

### 3.1 Main effect of childhood socioeconomic status

The influence of childhood SES on indulgent consumption behavior in adulthood has long been underexplored. Existing research provides inconsistent theoretical support for this issue. Some studies suggest that individuals raised in environments of scarcity may develop an instinct to resist temptation in order to maintain survival (Griskevicius et al., 2011), and thus exhibit greater resistance when faced with opportunities for indulgence. In contrast, individuals with higher childhood SES are more likely to view self-indulgence as a natural entitlement (Liu et al., 2019) that does not require restraint. On the other hand, some research indicates that lower childhood SES enhances individuals' sensitivity to opportunities (Proffitt Leyva et al., 2020), making them more likely to engage in choices that provide immediate gratification. Conversely, individuals with higher childhood SES and better education are thought to possess stronger self-control to resist temptation (Mittal and Griskevicius, 2014; Thompson et al., 2020). This study uses four experimental scenarios to simulate various decision-making contexts, confirming the significant positive effect of childhood SES on indulgent consumption. Interestingly, it was found that an individual's current SES does not significantly influence their indulgent consumption behavior. This finding further supports the idea that childhood SES, rather than current SES, is a more accurate predictor of behavioral patterns and preferences (Griskevicius et al., 2011; Zhang et al., 2020; Giroux et al., 2021). Both subjective and objective measures of childhood SES were employed in this study, yielding consistent results, which further indicates the robustness of this finding.

### 3.2 Mediating effect of pursuit of pleasure

In this study, we identified and confirmed the mediating role of the pursuit of pleasure in the positive effect of childhood SES on indulgent consumption. Compared to individuals with low childhood SES, those with high childhood SES are more likely to view themselves as independent and distinct from others, tending to feel more in control of their own destinies and more capable of shaping the world around them (Stephens et al., 2009). They also exhibit a stronger inclination toward self-gratification (Snibbe and Markus, 2005; Zhao et al., 2022). The pursuit of pleasure reflects an individual's motivation to seek novel rewards (Chen et al., 2015). Consequently, when faced with indulgent choices, individuals with higher childhood SES, under the influence of the pursuit of pleasure, are more inclined to seek immediate gratification. However, the sense of control is considered a key psychological mechanism driving behaviors associated with fast and slow life history strategies (Mittal and Griskevicius, 2014). Specifically, individuals with lower childhood SES tend to exhibit weaker self-control (Mittal and Griskevicius, 2014), and failures in self-control are more likely to trigger indulgent consumption (Kahn and Brian, 2004). Thus, in Study 4, we excluded the potential mediating role of sense of control in explaining the impact of childhood SES on indulgent consumption, further highlighting the explanatory effect of the pursuit of pleasure.

### 3.3 Moderating effect of sense of worthiness

This study constructed a moderated mediation model and confirmed, through Study 3, that the sense of worth positively moderates the mediating role of the pursuit of pleasure between childhood SES and indulgent consumption willingness. Specifically, as individuals' sense of worth increases, the mediating effect of the pursuit of pleasure between childhood SES and indulgent consumption becomes stronger; conversely, it weakens as the sense of worth decreases. The emergence of this moderating effect can be attributed to the idea that sense of worth serves as a special form of legitimizing justification (Heiland and Veilleux, 2022). Such legitimizing justifications can reduce the guilt associated with indulgent consumption (Haws and Liu, 2016), and the sense of worth provides consumers with more rationalizations to yield to the temptations of indulgent products.

### 3.4 Theoretical implications

First, this study provides a new theoretical perspective on the factors influencing indulgent consumption. Rather than limiting ourselves to the five types of influencing factors identified in the existing literature (Yang and Jin, 2018; Nenkov and Scott, 2014), we have explored childhood socioeconomic status (SES) as a more subtle and profound antecedent influence, drawing from life history theory. The results indicate that childhood SES positively influences indulgent consumption in adulthood, while the impact of current SES is not significant. This supports the existing literature suggesting that childhood SES has a stronger predictive power for behavior than current SES (Griskevicius et al., 2011; Zhang et al., 2020).

Second, this study extends the application of childhood SES. Existing research on childhood SES has largely focused on individuals' response patterns to their environment and the formation of traits (Griskevicius et al., 2011; Thompson et al., 2020), with limited attention to consumer behavior, particularly indulgent consumption. This study establishes a link between childhood SES and indulgent consumption, constructing a more comprehensive moderated mediation model. This provides a theoretical foundation for further exploration of the impact of childhood SES on other consumer behaviors.

### 3.5 Practical implications

The findings of this study contribute to a deeper understanding of consumer behavior and offer guidance for marketing practices. First, consumers need to recognize the subtle influence of childhood SES on their consumer behavior in adulthood. Consumers with higher childhood SES should be aware of their tendency toward indulgent consumption. When making consumption decisions, they should consciously reflect on whether a product is truly necessary, avoiding the excessive pursuit of pleasure that may undermine long-term wellbeing. Second, for businesses and marketing professionals, it is essential to challenge intuitive misconceptions when targeting potential markets for indulgent consumption. There should be a shift away from overemphasizing current factors such as income, spending power, and occupation, and instead, attention should be directed toward consumers' background and upbringing, as these factors have stronger predictive power for indulgent consumption. Finally, when promoting or selling indulgent products, it is advisable to emphasize the consumer's sense of worth. Strengthening the rationale for indulgent consumption decisions will stimulate desire and encourage purchases.

### 3.6 Limitations and future directions

First, this study's experimental design draws on representative decision-making scenarios from existing literature on indulgent consumption. The design of indulgent items was validated through manipulation checks, and the data analysis results supported our previous hypotheses. However, whether these conclusions hold in real-world consumer contexts remains uncertain, and the external validity of the findings requires verification through future field experiments.

Second, the measurement of childhood SES in this study primarily relied on participants' subjective recall of their childhood environment. In Study 4, by incorporating an objective measure of childhood SES, we found that subjective childhood SES provided a better prediction of indulgent consumption than objective childhood SES. While both measures are highly correlated, future research should carefully balance the trade-off between objective accuracy and predictive validity in behavioral decision-making. Identifying the “optimal balance” between these two approaches is an important issue that remains to be addressed.

Finally, this study has repeatedly demonstrated the positive effect of childhood SES on indulgent consumption through four experiments, underscoring the robustness of the findings. However, some “seemingly contradictory” results in existing research have not been sufficiently addressed. For example, research on uncertainty and risk management suggests that individuals with high childhood SES delay immediate gratification to increase future rewards, whereas those with low childhood SES prioritize immediate gains (Frankenhuis et al., 2016). Given that immediate gratification is closely related to indulgent consumption, future research should attempt to explore a new perspective that reconciles these two “opposite results,” allowing them to form a “unified opposition” within a larger theoretical framework.

## 4 Conclusion

This study aims to explore the impact of childhood SES on indulgent consumption and develops a comprehensive moderated mediation model. A total of 627 participants were recruited, and four experimental scenarios were used to systematically test six research hypotheses. The findings are as follows: (1) Childhood SES positively influences indulgent consumption in adulthood; specifically, individuals with higher childhood SES are more likely to engage in indulgent consumption compared to those with lower childhood SES. Interestingly, current SES does not significantly affect indulgent consumption. (2) The pursuit of pleasure mediates the relationship between childhood SES and indulgent consumption; that is, individuals with higher childhood SES are more likely to seek novelty and potential rewards, thereby yielding to the temptation of indulgent items. (3) Self-worth moderates the mediating effect of the pursuit of pleasure; when an individual's sense of worth is higher, the mediating effect of pleasure-seeking in the relationship between childhood SES and indulgent consumption is stronger, and vice versa. (4) The sense of control does not explain the impact of childhood SES on indulgent consumption behavior, suggesting that individuals with higher childhood SES engage in indulgent consumption not due to a greater sense of control over their external environment.

## Data Availability

The original contributions presented in the study are included in the article/supplementary material, further inquiries can be directed to the corresponding authors.

## References

[B1] Abell A. Biswas D. Mera C. A. (2024). Food and technology: using digital devices for restaurant orders leads to indulgent outcomes. J. Acad. Mark. Sci. 52, 1673–1691. 10.1007/s11747-024-01029-6

[B2] Ahuvia A. C. Wong N. Y. (2002). Personality and values based materialism: their relationship and origins. J. Consum. Psychol. 12, 389–402. 10.1016/S1057-7408(16)30089-4

[B3] Berry C. J. (1994). The idea of luxury: a conceptual and historical investigation. Am. Hist. Rev. 101, 449–450. 10.1017/CBO9780511558368

[B4] Biswas D. Szocs C. Chacko R. Wansink B. (2017). Shining light on atmospherics: how ambient light influences food choices. J. Marketing. Res. 54, 111–123. 10.1509/jmr.14.011511670861

[B5] Carey R. M. Markus H. R. (2016). Social class matters: a rejoinder. J. Consum. Psychol. 26, 599–602. 10.1016/j.jcps.2016.08.007

[B6] Cavanaugh L. A. (2014). Because I (don't) deserve it: how relationship reminders and deservingness influence consumer Indulgence. J. Marketing. Res. 51, 218–232. 10.1509/jmr.12.013311670861

[B7] Chen C. Hewitt P. L. Flett G. L. (2015). Preoccupied attachment, need to belong, shame, and interpersonal perfectionism: an investigation of the perfectionism social disconnection model. Pers. Indiv. Differ. 76, 177–182. 10.1016/j.paid.2014.12.001

[B8] Dambrun M. Ricard M. (2011). Self-centeredness and selflessness: a theory of self-based psychological functioning and its consequences for happiness. Rev. Gen. Psychol. 15, 138–157. 10.1037/a0023059

[B9] Dholakia U. M. Gopinath M. Bagozzi R. P. (2005). The role of desires in sequential impulsive choices. Organ. Behav. Hum. Dec. 98, 179–194. 10.1016/j.obhdp.2005.05.003

[B10] Fishbach A. Dhar R. (2005). Goals as excuses or guides: the liberating effect of perceived goal progress on choice. J. Consum. Res. 32:370–377. 10.1086/497548

[B11] Fishbach A. Zhang Y. (2008). Together or apart: when goals and temptations complement versus compete. J. Pers. Soc. Psychol. 94, 547–559. 10.1037/0022-3514.94.4.547 18361671

[B12] Frankenhuis W. E. Panchanathan K. Nettle D. (2016). Cognition in harsh and unpredictable environments. Curr. Opin. Psychol. 7, 76–80. 10.1016/j.copsyc.2015.08.011

[B13] Giroux M. Franklin D. Kim J. Park J. Kwak K. (2021). The impact of same versus different price presentation on travel choice and the moderating role of childhood socioeconomic status. J. Travel Res. 61, 674–695. 10.1177/0047287520988903

[B14] Griskevicius V. Tybur J. M. Delton A. W. Reboertson T. E. (2011). The influence of mortality and socioeconomic status on risk and delayed rewards: a life history theory approach. J. Pers. Soc. Psychol. 100, 1015–1026. 10.1037/a0022403 21299312 PMC3298774

[B15] Haws K. L. Liu P. J. (2016). Combining food type(s) and food quantity choice in a new food choice paradigm based on vicevirtue bundles. Appetite 103, 441–449. 10.1016/j.appet.2015.11.012 26585634

[B16] Heiland A. M. Veilleux J. C. (2022). Because you had a bad day: the role of negative affect and justification in self-control failure. Cogn. Emot. 36, 912–927. 10.1080/02699931.2022.2067134 35475950

[B17] Huberts J. C. D. W. Evers C. Ridder D. T. D. (2014). ‘Because I am worth it': a theoretical framework and empirical review of a justification-based account of self-regulation failure. Pers. Soc. Psychol. Rev. 18, 119–138. 10.1177/1088868313507533 24214148

[B18] Ibane-Aonso J. A. Company-Cordoba R. Garc de La Cadena C. Sianes A. Simpson I. C. (2021). How living in vulnerable conditions undermines cognitive development: evidence from the pediatric population of guatemala. Children 8:90. 10.3390/children8020090 33572817 PMC7912439

[B19] Infurna F. J. Gerstorf D. Ram N. Wagner G. G. (2011). Long-term antecedents and outcomes of perceived control. Psychol. Aging. 26, 559–575. 10.1037/a0022890 21517184 PMC3319760

[B20] Kahn B. E. Brian W. (2004). The influence of assortment structure on perceived variety and consumption quantities. J. Consum. Res. 30, 519–533. 10.1086/380286

[B21] Kasser T. Ryan R. M. Zax M. Sameroff A. J. (1995). The relations of maternal and social environments to late adolescents' materialistic and prosocial values. Dev. Psychol. 31, 907–914. 10.1037//0012-1649.31.6.907

[B22] Kathryn M. S. Staelin R. Huber J. (2008). Using extremeness aversion to fight obesity: policy implications of context dependent demand. J. Consum. Res. 35, 406–422. 10.1086/587631

[B23] Kivetz R. Zheng Y. H. (2006). Determinants of justification and self-control. J. Exp. Psychol. Gen. 135, 572–587. 10.1037/0096-3445.135.4.572 17087574

[B24] Kokkoris M. D. Hoelzl E. Alós-Ferrer C. (2019). True to which self? Lay rationalism and decision satisfaction in self-control conflicts. J. Pers. Soc. Psychol. 117, 417–447. 10.1037/pspp0000242 30920281

[B25] Kusserow A. S. (1999). Dehomogenizing American individualism: socializing hard and soft individualism in manhattan and queens. Ethos 27, 210–234. 10.1525/eth.1999.27.2.210

[B26] Li H. Song Y. Xie X. (2020). Altruistic or selfish? Responses when safety is threatened depend on childhood socioeconomic status. Eur. J. Soc. Psychol. 50, 1001–1016. 10.1002/ejsp.2651

[B27] Li Y. Zhang Y. Jiang Y. Li H. Mi S. Yi G. J.. (2008). Reliability and validity analysis of the Chinese version of the behavioral inhibition/activation system scale. Chin. J. Ment. Health 22:4.32457687

[B28] Liu Z. Sun X. Tsydypov L. (2019). Scarcity or luxury: which leads to adolescent greed? Evidence from a large-scale Chinese adolescent sample. J. Adolesc. 77, 32–40. 10.1016/j.adolescence.2019.10.002 31605887

[B29] Lloyd R. S. Faigenbaum A. D. Stone M. H. Oliver J. L. Jeffreys I. Moody J. A.. (2014). Position statement on youth resistance training: the 2014 international consensus. Brit. J. Sport. Med. 48, 498–505. 10.1136/bjsports-2013-092952 24055781

[B30] Luthar S. S. D'Avanzo K. (1999). Contextual factors in substance use a study of suburban and inner-city adolescents. Dev. Psychopathol. 11, 845–867. 10.1017/S0954579499002357 10624729 PMC3535189

[B31] Markus H. R. Conner A. (2013). Clash! 8 Cultural Conflicts That Make Us Who We Are. New York: Hudson Street Press.

[B32] May F. Irmak C. (2014). Listening indulgence in the present by distorting memories of past behavior. J. Consum. Res. 41, 624–641. 10.1086/676981

[B33] Miller A. L. Gearhardt A. N. Retzloff L. Sturza J. Kaciroti N. Lumeng J. C. (2018). Early childhood stress and child age predict longitudinal increases in obesogenic eating among low–income children. Acad. Pediatr. 18, 685–691. 10.1016/j.acap.2018.01.007 29357310 PMC6067997

[B34] Miller D. T. Effron D. A. (2010). Psychological license: when it is needed and how it functions. Adv. Exp. Soc. Psychol. 43, 115–155. 10.1016/S0065-2601(10)43003-8

[B35] Mittal C. Griskevicius V. (2014). Sense of control under uncertainty depends on People's childhood environment: a life history theory approach. J. Pers. Soc. Psychol. 107, 621–637. 10.1037/a0037398 25133717

[B36] Moeller F. G. Barratt E. S. Dougherty D. M. Schmitz J. M. Swann A. C. (2001). Psychiatric aspects of impulsivity. Am. J. Psychiat. 158, 1783–1793. 10.1176/appi.ajp.158.11.1783 11691682

[B37] Nenkov G. Y. Scott M. L. (2014). So cute I could eat it up: priming effects of cute products on indulgent consumption. J. Consum. Res. 41, 326–341. 10.1086/676581

[B38] O'Brien E. Roney E. (2017). Worth the wait? Leisure can be just as enjoyable with work left undone. Psychol. Sci. 28, 1000–1015. 10.1177/0956797617701749 28590810

[B39] Odgers C. L. Moffitt T. E. Broadbent J. M. (2008). Female and male antisocial trajectories: From childhood origins to adult outcomes. Dev. Psychol. 20, 673–716. 10.1017/S0954579408000333 18423100

[B40] Park J. Kim J. Lee D. C. Kim S. S. Voyer B. G. Kim C.. (2022). The impact of COVID-19 on consumer evaluation of authentic advertising messages. Psychol. Market. 39, 76–89. 10.1002/mar.21574 34539052 PMC8441704

[B41] Proffitt Leyva R. P. Mengelkoch S. Gassen J. Ellis B. J. Russell E. M. Hill S. E. (2020). Low socioeconomic status and eating in the absence of hunger in children aged 3–14. Appetite 154:104755. 10.1016/j.appet.2020.104755 32579973

[B42] Salerno A. Laran J. Janiszewski C. (2014). Hedonic eating goals and emotion: when sadness decreases the desire to indulge. J. Consum. Res. 41, 135–151. 10.1086/675299

[B43] Snibbe A. C. Markus H. R. (2005). You can't always get what you want: educational attainment, agency, and choice. J. Pers. Soc. Psychol. 88, 703–720. 10.1037/0022-3514.88.4.703 15796669

[B44] Stamos A. Altsitsiadis E. Dewitte S. (2019). Investigating the effect of childhood socioeconomic background on interpersonal trust: Lower childhood socioeconomic status predicts lower levels of trust. Pers. Indiv. Differ. 145, 19–25. 10.1016/j.paid.2019.03.011

[B45] Stephens N. M. Hamedani M. G. Markus H. R. Bergsieker H. B. Eloul L. (2009). Why did they “choose” to stay? Perspectives of hurricane katrina observers and survivors. Psychol Sci. 20, 878–886. 10.1111/j.1467-9280.2009.02386.x 19538433

[B46] Taylor C. Webb T. L. Sheeran P. (2014). ‘I deserve a treat!': justifications for indulgence undermine the translation of intentions into action. Bri. J. Psychol. Soc. 53, 501–520. 10.1111/bjso.12043 23855962

[B47] Thompson D. V. Hamilton R. W. Banerji I. (2020). The effect of childhood socioeconomic status on patience. Organ. Behav. Hum. Dec. 157, 85–102. 10.1016/j.obhdp.2020.01.004

[B48] Vanbergen N. Laran J. (2016). Loss of control and self regulation: the role of childhood lessons. J. Consum. Res. 43, 534–548. 10.1093/jcr/ucw042

[B49] Weininger E. B. Lareau A. LaRossa R. (2009). Paradoxical pathways: an ethnographic extension of Kohn's findings on class and childrearings. J. Marriage. Fam. 71, 680–695. 10.1111/j.1741-3737.2009.00626.x

[B50] Wen Z. L. Ye B. J. (2014). Different methods for testing moderated mediation models: competitors or backups? Acta. Psychol. Sinica. 46, 714–726. 10.3724/SP.J.1041.2014.0071437113526

[B51] Yang S. G. Jin L. Y. (2018). A review of research on the consumption behavior of indulgences and self-regulated things. Foreign Econ. Manag. 40, 84–97.

[B52] Zhang Z. Liu H. Choi S. W. (2020). Early-life socioeconomic status, adolescent cognitive ability, and cognition in late midlife: evidence from the wisconsin longitudinal Study. Soc. Sci. Med. 244, 1–40. 10.1016/j.socscimed.2019.112575 31606188 PMC6926157

[B53] Zhao J. Childers C. Sang H. Cheng J. Vigo R. (2022). The effect of anger on variety seeking for consumers of differing socio-economic backgrounds. Curr. Psychol. 40, 1–8. 10.1007/s12144-019-00476-7

